# A Blood‐Responsive AIE Bioprobe for the Ultrasensitive Detection and Assessment of Subarachnoid Hemorrhage

**DOI:** 10.1002/advs.202205435

**Published:** 2023-01-22

**Authors:** Maliang Tao, Jian Mao, Yun Bao, Fan Liu, Yiying Mai, Shujuan Guan, Shihua Luo, Yifang Huang, Zixiong Li, Yuan Zhong, Binbin Wei, Jun Pan, Qian Wang, Lei Zheng, Bo Situ

**Affiliations:** ^1^ Department of Laboratory Medicine Nanfang Hospital Southern Medical University Guangzhou 510515 China; ^2^ Guangdong Engineering and Technology Research Center for Rapid Diagnostic Biosensors Nanfang Hospital Southern Medical University Guangzhou 510515 China; ^3^ Department of Neurosurgery Nanfang Hospital Southern Medical University Guangzhou 510515 China; ^4^ The Second Clinical College Southern Medical University Guangzhou 510515 China; ^5^ Center for Clinical Laboratory Diagnosis and Research the Affiliated Hospital of Youjiang Medical University for Nationalities Baise 533000 China; ^6^ Department of Clinical Laboratory the First Affiliated Hospital of Guangxi Medical University Nanning 530021 China

**Keywords:** aggregation‐induced emission, biological probe, endovascular perforation model, strokes, subarachnoid hemorrhage

## Abstract

Subarachnoid hemorrhage (SAH) is a severe subtype of stroke caused by the rupturing of blood vessels in the brain. The ability to accurately assess the degree of bleeding in an SAH model is crucial for understanding the brain‐damage mechanisms and developing therapeutic strategies. However, current methods are unable to monitor microbleeding owing to their limited sensitivities. Herein, a new bleeding assessment system using a bioprobe TTVP with aggregation‐induced emission (AIE) characteristics is demonstrated. TTVP is a water‐soluble, small‐molecule probe that specifically interacts with blood. Taking advantage of its AIE characteristics, cell membranes affinity, and albumin‐targeting ability, TTVP fluoresces in bleeding areas and detects the presence of blood with a high signal‐to‐noise (S/N) ratio. The degree of SAH bleeding in an endovascular perforation model is clearly evaluated based on the intensity of the fluorescence observed in the brain, which enables the ultrasensitive detection of mirco‐bleeding in the SAH model in a manner that outperforms the current imaging strategies. This method serves as a promising tool for the sensitive analysis of the degree of bleeding in SAHs and other hemorrhagic diseases.

## Graphical abstract

An ultrasensitive blood‐responsive probe with unique features for detecting blood and assessing bleeding in a mouse model of subarachnoid hemorrhage (SAH) is presented. By exploiting its aggregation‐induced emission (AIE) characteristics, cell membrane affinity, and albumin‐targeting ability, the bleeding degree of SAH can be sensitively evaluated from the intensity of the fluorescence observed in the brain.

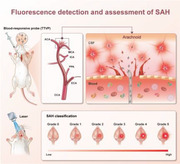

## 1 Introduction

Stroke is a leading cause of permanent disability and death worldwide. As a type of stroke, subarachnoid hemorrhage (SAH), involves bleeding within the subarachnoid cavity and is caused by a ruptured vessel on the surface of brain.^[^ ^1^, ^2^, ^3^ ^]^ SAH is a life‐threatening event, with ≈30% of patients with SAH within one mouth of onset,^[^ ^4^, ^5^ ^]^ and those who survive show varying degrees of brain damage that often lead to long‐term or even permanent coma, cognitive impairment, neurological dysfunction, and disability.^[^ ^6^, ^7^, ^8^ ^]^ Since the pathogenesis of brain injury caused by SAH remains largely unclear, effective treatment strategies are still lacking.

Animal models are crucial for studying human diseases and developing therapeutic strategies.^[^ ^9^, ^10^ ^]^ Currently, the endovascular perforation model is the most widely used method for investigating SAH. In this model, SAH is induced by puncturing the intracranial vessels of a mouse with a thin thread.^[^ ^11^, ^12^, ^13^ ^]^ However, the amount of intracranial hemorrhage in a mouse is often difficult to determine unless it is euthanized and dissected, owing to the lack of guidance provided by effective imaging. Using such a model to monitor the severity, progression, and recovery of SAH is challenging. Although the use of computed tomography (CT) and magnetic resonance imaging (MRI) has been reported,^[^ ^14^, ^15^ ^]^ their performance is far from satisfactory. Trace hemorrhages that occur in mouse brains (less than 10 µL) are difficult to discern owing to the limited resolution. Hence, the development of a sensitive hemorrhage‐imaging approach is highly desirable given the abovementioned limitations.

Fluorescence imaging is an ideal strategy for observing SAH in mice because it can sensitively identify microlesions and biological processes,^[^ ^16^, ^17^, ^18^ ^]^ however, while developing a bleeding‐responsive fluorescence probe is key, it is nevertheless challenging. Such probes need to be hypotoxic, specifically activated by blood, and correlate with the amount of bleeding. In addition, because hemoglobin, the most abundant protein in blood strongly absorbs almost all visible light,^[^ ^19^, ^20^ ^]^ the designed probe needs to emit in the near‐infrared (NIR) region with a high signal‐to‐noise (S/N) ratio. Unfortunately, a fluorescence probe that possesses all of these required properties has yet to be reported.^[^ ^21^ ^]^

**Figure 1:**
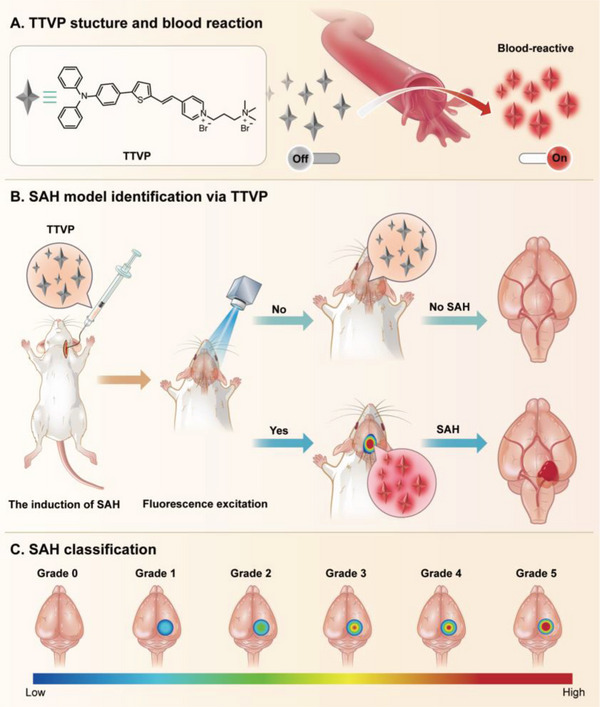
Schematic illustrating the use of the NIR blood‐responsive AIE probe for SAH detection and classification. A) TTVP structure and blood reaction. The turn‐on pattern of the AIE probe after interacting with whole blood. B) Mouse model for identifying SAH using TTVP. C) SAH classification based on the intensity of the fluorescence observed in the brain.

Herein, we describe an ultrasensitive bleeding assessment system using a bioprobe TTVP by taking advantage of its unique properties (**Figure** **1**). TTVP does not emit in aqueous solution but emits bright red fluorescence in blood solutions, which is ascribable to its aggregation‐induced emission (AIE)^[^ ^22^, ^23^, ^24^, ^25^ ^]^ features, cell membrane affinity, and albumin‐targeting ability. This system effectively grades the degree of bleeding in the SAH model with high S/N ratios based on the intensity of the fluorescence observed in the brain, which greatly increases microbleeding assessment sensitivity. To the best of our knowledge, this is the first report that describes using molecular aggregation to light‐up blood in the NIR region in an SAH model, and serves as an alternative visualization and bleeding‐assessment tool that augments imaging approaches used in SAH disease research.

## 2 Results

### 2.1 Photophysical Characteristics and Blood‐Responsive Properties of TTVP

The small‐molecule bioprobe TTVP was chosen after screening the AIE molecular library. TTVP was prepared according to the synthetic route presented in Scheme S1 (Supporting Information), and its structure was fully characterized and verified by NMR spectroscopy and HRMS measurements (Figures S1–S3, Supporting Information). It consists of a prototypical 4‐bromo‐N, N‐diphenylaniline AIE unit and pyridinium groups.

**Figure 2:**
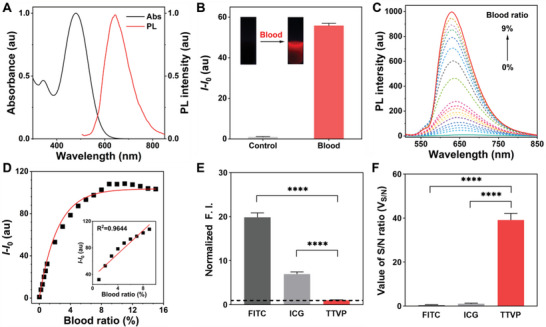
Photophysical and blood‐responsive properties of TTVP. A) Normalized absorption and PL spectra of TTVP in aqueous solution. B) TTVP is significantly lit‐up by the addition of blood (5 µL). Inset: fluorescence images of TTVP in PBS before and after the addition of blood under 365 nm UV light. C) Fluorescence titration of TTVP by the stepwise addition of blood (0 to 9%) in PBS (pH = 7.4). D) Calibration curve of PL intensity at 645 nm as a function of blood concentration (0 to 15%); *I*_0_ = PL intensity in the absence of blood. Inset: linear region of the TTVP‐to‐blood binding isotherm. E) Normalized background fluorescence intensities (F.I.s) of FITC, ICG, and TTVP in the absence of blood. (F) S/N ratios (*V*_S/N_) of FITC, ICG, and TTVP after incubation with 1% blood solution. Data are presented as means ± SDs (*n* = 3 per group); ****p < 0.0001. [Probe] = 100 × 10^−6^ m.

The photophysical properties of TTVP were next investigated; it exhibits an absorption maximum at 480 nm and an emission peak at 645 nm in aqueous solution (**Figure** **2**A). Photoluminescence spectra were then acquired in THF/water mixtures. TTVP is almost nonemissive in aqueous solution, which is mainly ascribable to the rotational motions of its molecular rotors that consume exciton energy and increase nonradiative decay rates, leading to none emission.^[^ ^26^ ^]^ The photoluminescence (PL) intensities gradually increased as the THF fraction (*f*_T_) was increased from 0 to 99% owing to the formation of nanoaggregates (Figure S4, Supporting Information). The strongest PL was observed for aggregates produced in 99% THF, in which an ≈128.6‐fold increase in PL was observed compared with that of the aqueous solution. This significantly enhanced emission is possibly attributable to the restricted rotatory motions in the aggregated state, which highlights the AIE properties of TTVP.

We next investigate whether TTVP was responsive to blood or not. Consistent with previous findings, TTVP is completely soluble in PBS and essentially nonemissive (Figure 2B). However, significantly enhanced fluorescence was observed by the naked eye under UV light when a drop of blood was added (Figure S5 and Movie S1, Supporting Information). This process required only 20 s to emit 80% of the maximum fluorescence intensity, and plateaued in ≈10 min, highlighting the rapid detection ability of TTVP (Figure S6, Supporting Information). TTVP stability was evaluated using biological samples. Figure S7 (Supporting Information) shows that the fluorescent state of TTVP was almost unaffected as the pH was increased from 3 to 12; it was also stable in real cerebrospinal fluid (CSF) over 24 h, which is crucial for long‐term imaging in complex biological environments.^[^ ^27^, ^28^ ^]^ These results suggest that TTVP can serve as a simple, rapid, and stable probe for detecting hemorrhages.

### 2.2 Ultrahigh Signal‐to‐Noise (S/N) Ratio of TTVP for Blood Detection

The distinctive properties of TTVP prompted us to explore its performance as a blood indicator. We first studied the relationship between PL intensity and blood concentration. As shown in Figure 2C, TTVP PL intensities increased continuously with the increasing blood concentration, with the highest intensity observed at a 9% blood concentration upon aggregation, in which the PL intensity was enhanced to a factor of 108 compared with that observed in aqueous solution (Figure 2D). The significantly enhanced AIE is attributable to the restrict rotatory motions that activate radiative decay.^[^ ^29^ ^]^ The inset plot in Figure 2D shows a linear relationship between intensity and blood concentration in the 1–9% range. Notably, a fluorescence signal was detected at low blood volume (1 µL), highlighting the good sensitivity of TTVP for blood detection.

We then evaluated the initial background and signal amplification of TTVP, with the results obtained compared with those of indocyanine green (ICG) and fluorescein isothiocyanate (FITC), the most commonly used clinical probes.^[^ ^30^, ^31^, ^32^ ^]^ TTVP exhibited a very low background signal (1/20th and 1/7th those of FITC and ICG, respectively) in aqueous solution in the absence of blood (Figure 2E), which is ascribable to its high hydrophilicity and AIE properties (Figure S8, Supporting Information). Moreover, TTVP showed an excellent S/N ratio (*V*_S/N_ = 39) when interacting with blood, far exceeding those of FITC (*V*_S/N_ = 0.5) and ICG (*V*_S/N_ = 1.1) (Figure 2F); this significant S/N ratio is attributable to the low initial background signal and switchable fluorescence properties of TTVP. These remarkable characteristics of TTVP make it an ideal candidate as an ultrasensitive and high‐contrast agent for the detection of blood.

### 2.3 In Vitro Mechanistic Studies of TTVP Interacting with Blood

**Figure 3:**
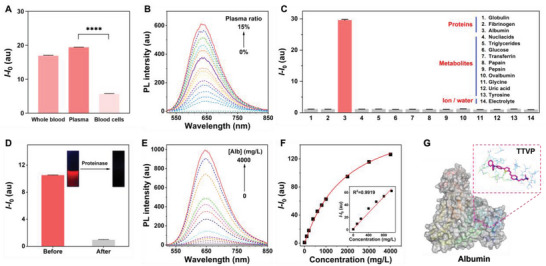
In vitro mechanistic studies of TTVP for blood detection. A) Responsiveness of TTVP toward whole blood, plasma and blood cells respectively. Blood cells and plasma were separated from 1 mL whole blood and added with an equal volume of PBS to 1 mL; [Blood] = 3%. Data are presented as means ± SDs (*n* = 3 per group); ****p < 0.0001. B) Fluorescence titration of TTVP by the stepwise addition of plasma (0 to 15%) in PBS (pH = 7.4). C) Binding ability of TTVP toward various components of plasma. Components 1–5 are normalized against a blood concentration of 1%; Components 6–14 are normalized to 100 mg mL^−1^. D) Effect of proreinase K on the TTVP/blood interactons. [Proteinase K] = 4 mg mL^−1^. E) PL spectra of TTVP in PBS containing various concentrations of albumin (0‐4000 mg L^−1^). F) Calibration curve of PL intensity at 645 nm as a function of albumin concentrations; *I*_0_ = PL intensity in the absence of albumin. Inset: linear region of the TTVP‐to‐albumin binding isotherm (0‐1000 mg L^−1^). G) Molecular docking analysis of TTVP/albumin binding site by PYMOL.

In vitro specificity studies were used to determine which part of the blood is responsible for lighting up TTVP. Considering the complexity of blood components, whole blood was divided into blood cells and plasma. As shown in **Figure** **3**A, TTVP showed a 26‐fold fluorescence enhancement on blood cells at a blood concentration of 3%, which was consistent with the previously reported affinity of TTVP for cell membranes.^[^ ^26^ ^]^ Notably, TTVP showed a significant fluorescence response mainly to plasma compared with blood cells (Figure 3B and Figures S9 and S10, Supporting Information). Further subdivision of plasma revealed that TTVP mainly responded to albumin rather than blood metabolites and ions (Figure 3C).^[^ ^33^, ^34^, ^35^ ^]^ To further verify this effect, we used proteinase K to metabolize the plasma proteins, especially albumin. As displayed in Figure 3D, the fluorescence generated by blood interactions was almost absent after proteinase digestion, confirming our previous findings. The specific relationship between TTVP and albumin level was also explored. In a similar manner, TTVP showed a good response to albumin concentrations in the 0–4000 mg L^−1^ range, with a linear coefficient of determination (*R* ^2^) of 0.9916 observed in the 0–1000 mg L^−1^ range (Figures 3E,F). Moreover, the spatial conformations determined by molecular docking reveal that TTVP is likely to enter the hydrophobic pockets of albumin as binding sites, which switches on its fluorescence state (Figure 3G). These results clearly suggest that TTVP is highly specific toward albumin and cell membranes and is capable of selectively detecting blood.

### 2.4 In Vivo NIR Fluorescence Imaging of SAH Induction

**Figure 4:**
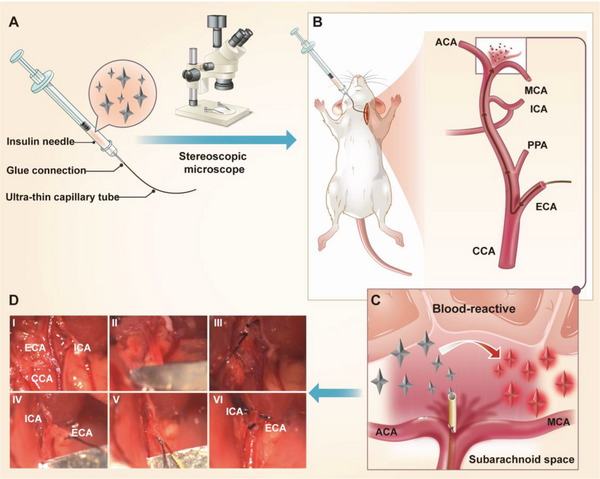
Modified endovascular‐perforation‐based SAH‐induction strategy. A) Depicting the SAH‐induction working principle. TTVP is loaded into a syringe and injected into the subarachnoid space of a mouse during SAH induction. B) The structure of the new injecting device; it consists of an insulin needle and an ultra‐thin capillary tube (0.01 mm), which are tightly glued together. C) Anatomical diagram of the brain base of a mouse under a stereo microscope after puncturing the ACA. a) saddle back; b) intracranial segment of the right internal carotid artery; c) hypothalamus; d) intracranial segment of the left internal carotid artery. D) Stereomicroscopy views of the SAH‐induction process. I) Dissociate the CCA, ICA, and ECA. II) Clamp the CCA. III) Ligate the proximal and centrifugal ends of the ECA separately. IV) Cut the blood vessel between the two ligation sites of the ECA, and pull the cord near the heart to form a straight line between the remaining ECA and ICA. V) Insert the capillary from the ECA to the intracranial segment of the ICA, and finally pierce the blood vessel and simultaneously inject TTVP. VI) Withdraw the capillary from the blood vessel and ligate the ECA rupture. CCA: common carotid artery; ICA: internal carotid artery; ECA: external carotid artery; PPA: paramedian pontine arteries; MCA: middle cerebral artery; ACA: anterior communicating artery.

Having confirmed the blood‐driven specific NIR light‐up response in vitro, we next sought to evaluate its applicability to live animal imaging. Prior to any in vivo assessment, the cytotoxicity of TTVP was evaluated using the cell counting Kit‐8 (CCK‐8) cell proliferation assay. As shown in Figure S11 (Supporting Information), cell growth was not significantly affected by the addition of up to 50 µg mL^−1^ TTVP, which is indicative of low toxicity. We next examined the tissue penetrability of TTVP. The fluorescence signal was clearly detected through 0.75 cm thick chicken tissue (Figures S12 and S13, Supporting Information), which is mainly ascribable to the unique advantage of NIR luminescence.^[^ ^36^ ^]^ Endovascular perforation, one of the most common SAH induction methods, was used as a test model.^[^ ^11^ ^]^ To avoid direct contact with blood, a new injecting device was developed to bring TTVP directly into the subarachnoid spaces of mice during SAH induction. Aided by a stereo microscope, the ultrathin and highly flexible capillary tube of the intubation device was used to smoothly puncture the anterior communicating artery (ACA) in the Willis circle in the brain, with the TTVP probe simultaneously injected into the subarachnoid space for hemorrhage imaging (**Figure** **4**A–C). Moreover, the entire modeling process was recorded using a stereo microscope (Figure 4D), which demonstrates the feasibility of this modified strategy.

**Figure 5:**
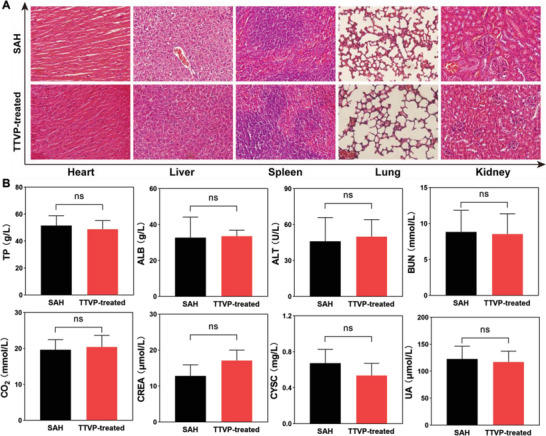
A) Microscopy images of tissue sections from SAH and TTVP‐treated SAH mice. B) Hepatic/renal function analyses of SAH and TTVP‐treated SAH mice. [TTVP] = 100 × 10^−6^ m. Data are presented as means ± SDs (*n* = 3 per group); ns indicated of none significance. TP: total protein; ALB: albumin; ALT: alanine aminotransferase; BUN: blood urea nitrogen; CO_2_: carbon dioxide; CREA: creatinine; CYSC: cystatin C; UREA: urea.

To further demonstrate that TTVP is biosafe in vivo, histological sections of major organs were stained with hematoxylin and eosin (H&E) to evaluate the effect of TTVP on SAH in mice. **Figure** **5**A and Figure S14 (Supporting Information) reveal that no noticeable damage or inflammatory lesions were observed, suggestive of low in vivo toxicity. Similar results were observed when neurological function was assessed (Figure S15 and Table S2, Supporting Information). Further hepatic and renal function analyses revealed that the TTVP‐treated mice showed no abnormalities (Figure 5B). The excellent tissue penetrability and good biocompatibility of TTVP suggest that it is well‐suited for in vivo imaging applications.

**Figure 6:**
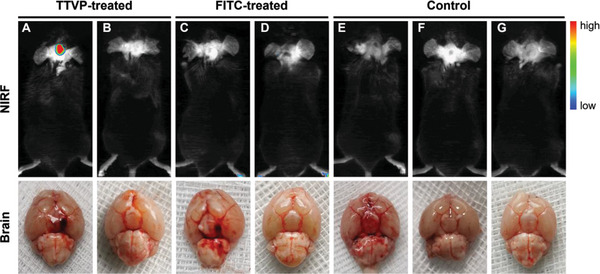
Representative near‐infrared fluorescence (NIRF) images and corresponding photographic images of the basal brains of various groups of mice. (A) Operation group of TTVP‐treated SAH mice. B) Sham operation group of TTVP‐treated SAH mice. C) Operation group of FITC‐treated SAH mice. D) Sham operation group of FITC‐treated SAH mice. E) Normal operation group of SAH mice. F) Normal sham operation group of SAH mice. G) Normal control mice. [Probe] = 100 × 10^−6^ m.

TTVP and FITC were “arterially injected” into different groups of C57 mice to observe fluorescence signals from their brains following SAH induction. **Figures** **6**A,B show that we are able to easily distinguish bleeding sites (with stronger red fluorescence signals) from the surrounding areas in the brains of the TTVP‐treated SAH mice, which were subsequently identified from the corresponding postmortem photographic images of their basal brains. In contrast, the FITC‐treated SAH mice showed no signals owing to the limited tissue penetration of non‐NIR light and the lack of blood reactivity (Figures 6C,D). A sham operation group was used to simulate a situation in which the intracranial blood vessel failed to puncture during the SAH induction process (Figures 6E–G). In this case, we confirmed that TTVP was transported to the peripheral blood and was completely metabolized within 24 h (Figure S16, Supporting Information), which is mainly ascribable to its small molecular mass and high hydrophilicity. Compared with traditional fluorescence probes, TTVP is better able to penetrate tissue and exhibits unique AIE properties that remarkably “turn‐on” fluorescence through interactions with blood.

### 2.5 New Grading System Based on Fluorescence Intensity for Assessing SAH Bleeding

**Figure 7:**
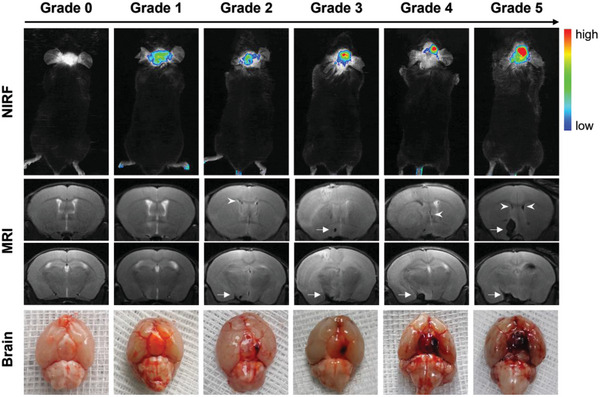
Representative fluorescence, T2‐weighted MR, photographic images of basal brains in each fluorescence grade. Arrows indicate basal subarachnoid hemorrhages (SAHs) clots and arrowheads indicate intraventricular hemorrhages in lateral ventricles.

We next systematically evaluated the further use of TTVP for grading SAH in vivo. An SAH model of unknown degree was then assessed using NIR fluorescence (NIRF) imaging. **Figure** **7** shows obvious fluorescence differences in the brains of SAH mice, which indicates that bleeding needs to be accurately assessed after inducting SAH. Based on the relative fluorescence signal in the brain, we stratified the degree of SAH into six grades: no bleeding (grade 0), diffuse bleeding or microbleeding (grade 1), minor bleeding (grade 2), small bleeding (grade 3), moderate bleeding (grade 4), and heavy bleeding (grade 5).

MRI and brain anatomy were used to further examine the reliability of this new fluorescence grading system for assessing hemorrhaging. Consistent with the NIRF imaging results, irregular black areas of varying sizes were observed in the circles of Willis and lateral ventricles of SAH mice, which implied the occurrence of intraventricular hemorrhage and the formation of blood clots. Similar results were observed in the anatomical photographic images (Figure 7). Interestingly, while fluorescence signals were clearly observed in the brains of some mice that exhibited diffuse or microbleeding, such signals were not observed by MRI (Figure 7). This observation is possibly attributable to the fact that TTVP interacts with albumin and blood cells in the blood at the molecular level, leading to higher sensitivity for the detection of microbleeding. A Comparison of different evaluation systems suggests that TTVP may serve as a powerful and sensitive tool for both identifying subarachnoid hemorrhaging and assessing its severity in vivo.

## 3 Discussion

The development of sensitive blood‐responsive probes is critical for detecting and assessing the degree of subarachnoid hemorrhaging. In this work, the AIE probe TTVP was found to emit significant NIR fluorescence in the presence of blood, with its fluorescence state switched on by interacting with blood, which demonstrates its great potential as an ultrasensitive blood indicator probe. In vitro studies revealed the ability of TTVP to detect blood is mainly achieved through the interaction with albumin and partly through the affinity to cell membranes. Notably, TTVP also showed great responsiveness to hemoglobin. However, free hemoglobin in peripheral blood would be bound by haptoglobin and degraded in the liver, thus we did not further explore the mechanism of TTVP‐to‐hemoglobin. This could be a potential way for detecting blood metabolism.

The administration method impacts the in vivo imaging performance and biological application of probes in a far‐reaching manner.^[^ ^37^, ^38^ ^]^ Traditional contrast agents are usually administered orally or through intravenous injection, irrespective of whether they are used for radionuclide or fluorescence imaging.^[^ ^39^, ^40^, ^41^ ^]^ Taking side effects into account, in this study we designed an injectable intubation device that delivers TTVP directly into the subarachnoid spaces of mice, which effectively realizes “arterial administration” in SAH mice. This administration route not only avoids direct contact between the probe and peripheral blood but also greatly reduces the potential influence of the probe on important mouse organs.^[^ ^42^, ^43^, ^44^ ^]^ Unfortunately, this method was unable to identify bleeding in mice that had already developed SAH. Intrathecal injection of TTVP may be a way of compensating this defect, and represents an important direction for subsequent optimization research;^[^ ^45^, ^46^ ^]^ it also provides new inspiration for further applications of the TTVP bleeding‐indicator probe, which may be extended to various internal hemorrhage models with a closed internal environment using different delivery methods.

In vivo imaging experiments revealed that TTVP is capable of visualizing and identifying the induction of SAH owing to its NIR luminescence and blood‐responsive properties. A system for visually grading bleeding was established based on fluorescence intensity in the brain. Our findings show that the introduced fluorescence‐grading system is superior to MRI for detecting microbleeding in SAH mice, which is attributable to two advantages. On the one hand, the unique luminescence mechanism of TTVP provides very high target‐to‐background signal ratios, which enables the sensitive detection of microbleeding. On the other hand, blood contains almost 180‐times more albumin than CSF, which is ascribable to the presence of the blood‐brain barrier, leading to ultrasensitive performance for the detection of bleeding.^[^ ^47^, ^48^ ^]^ Unfortunately, subsequent TTVP metabolism in the CSF needs to be further studied and discussed. Nevertheless, potential radioactive rays and contrast agents used in CT/MRI were shown not to affect our fluorescence grading system, which is also more sensitive for quantitatively assessing the relative degree of bleeding and SAH severity. Given the serious harm of stroke to human health, this new fluorescence‐based strategy for hemorrhage imaging would be a significant tool for better understanding the mechanism as well as the assessment of treatment effect of SAH. In addition, this method can lead to many new avenues of research on other hemorrhagic diseases.

## 4 Conclusion

An ultrasensitive fluorescence grading system based on the AIE probe TTVP was developed for detecting bleeding and assessing subarachnoid hemorrhages. The SAH mice model evaluated by this strategy not only retains its pathogenic characteristics consistent with SAH caused by ruptured aneurysms, but also can be used for comparison between individuals and grouped according to the degree of hemorrhage and SAH severity for subsequent mechanism research. To the best of our knowledge, this is the first time that a fluorescence‐based in vivo identification method and quantitative grading system has been established for assessing SAH bleeding, which is expected to play an essential role in further investigations into pathological mechanisms and novel therapeutic therapies. In future studies, we expect to use TTVP as an in situ bleeding indicator in other animal models of internal bleeding diseases, such as upper gastrointestinal bleeding and intestinal bleeding.

## 5 Experimental Section

### Materials and methods

Fetal bovine serum (FBS) and Dulbecco's modified Eagle medium (DMEM) were purchased from Gibco. Cell Counting Kit‐8 (CCK‐8) was purchased from Dojindo. Tetrahydrofuran (THF), dimethyl sulfoxide (DMSO), fluorescein isothiocyanate (FITC, 97%), indocyanine green (ICG, 98%), protease K (≥30 U mg^−1^), papain(>200 U mg^−1^), pepsin (>3000 U mg^−1^), human serum albumin (BR, 96–99%), globulin (BR, 96%), hemoglobin (BR, 98%), fibrinogen (BR, 90%), glucose (BR, 98%), transferrin (BR, 98%), ovalbumin (BR, 98%), glycine (BR, 98%), uric acid (BR, 99%), tyrosine (BR, 99%), triglycerides (BR, 98%), and all other chemicals and solvents used in this study were obtained from Sigma‐Aldrich or Macklin and used as received. NMR spectra were acquired on a Bruker Avance 600 MHz spectrometer against tetramethylsilane as the internal standard. Mass spectra were recorded using a Thermo Fisher Orbitrap Fusion Tribrid mass spectrometer, and UV–vis absorption spectra were obtained using a Shimadzu UV‐2600 spectrophotometer. Particle sizes were measured using a Malvern Zetasizer Nano S90 instrument. Photoluminescence (PL) spectra were recorded on a Perkin‐Elmer LS 55 spectrofluorometer.

### Synthesis of TTVP

TTVP was synthesized as previously described.^[^ ^26^ ^] 1^H NMR (600 MHz, CD_3_OD) *δ* 8.82 (d, *J* = 5.5 Hz, 2H), 8.15–8.11 (m, 3H), 7.58–7.54 (m, 2H), 7.48–7.46 (m, 1H), 7.38–7.35 (m, 1H), 7.32–7.28 (m, 4H), 7.08–7.07 (m, 7H), 7.02–6.98 (m, 2H), 4.64–4.62 (m, 2H), 3.60–3.58 (m, 2H), 3.22– 3.21 (m, 9H), 2.61–2.55 (m, 2H). ^13^C NMR (150 MHz, CD_3_OD) *δ* 154.4, 149.2, 148.6, 147.2, 147.1, 146.0, 143.7, 138.7, 134.2, 129.2, 126.7, 126.5, 124.8, 123.6, 123.4, 123.3, 122.2, 120.1, 62.5, 56.4, 52.6, 24.7; HRMS (ESI): calcd for C_35_H_37_N_3_S [M – 2Br]^2+^: 531.2697, found: 531.2698.

### Solution Preparation

TTVP, FITC and ICG were first dissolved in DMSO to prepare stock solution (10 × 10^−3^ m) and then added into PBS (pH = 7.40) with a final concentration of 100 × 10^−6^ m respectively; 10 mg of papain, pepsin, human serum albumin, globulin, fibrinogen, glucose, transferrin, ovalbumin, glycine, uric acid, tyrosine and triglycerides were dissolved in PBS solutions at 100 mg mL^−1^ respectively; Fresh PBS buffers of pH 3–12 were adjusted using sodium hydroxide (NaOH, 6 M) or hydrochloric acid (HCL, 6 M), and then measured by pH meter PHS‐25 (LEICI, Shanghai).

### Evaluating Cell Viability

Human umbilical vein endothelial cells (HUVECs) were cultured overnight in a 96 well‐plate at density of 5000 cells per well. After overnight culture, DMEM with various concentrations of TTVP was added to the plate and incubated for 4 h. The wells were washed with PBS and 10% CCK‐8 in DMEM (100 µL) was added to each well. The wells were incubated at 27°C for 1 h, after which the absorbance of each well was recorded at 450 nm using a microplate reader (Perkin‐Elmer Victor3^TM^).

### Tissue‐penetration Studies

TTVP solution (100 × 10^−6^ m, 200 µL) was placed into the wells of a black 96‐well plate. Chicken tissue of required thickness was overlaid on the bottom of each well and fluorescence images were acquired (excitation wavelength: 470 nm; emission wavelength: 600 nm; acquisition time: 60 s).

### Inducting SAH

All experiments conformed to the Guide for the Care and Use of Laboratory Animals published by the US National Institutes of Health (NIH Publication No. 85‐23, revised 1996) and were approved by the Animal Experimental Committee of Nanfang Hospital, Southern Medical University (AEC number: NFYY‐2016‐107). Eight‐week‐old male C57Bl/6J mice weighing 18–22 g were purchased from the Guangzhou Yancheng Biotechnology Co., Ltd., and kept in small groups under controlled temperature and humidity conditions. SAH was induced by single surgery using the previously described endovascular perforation technique.^[^ ^11^, ^12^ ^]^ A modified injecting device was used to puncture the ACA in the brain instead of the blunted 6–0 monofilament thread used in the traditional method (Figure 3).

### Establishing of Different Models in Living Mice

Mice were randomly selected for SAH induction and treated with TTVP or FITC (15 × 10^−3^ m, 2 µL DMSO). Probes were loaded and injected directly into the subarachnoid space of each mouse using the modified injecting device at the same time that the ACA in the brain was punctured. The sham operation group was induced in the same way as the operation group without puncturing the intracranial blood vessel. The control group was untreated. Neurological scoring, imaging, and blood sampling were conducted at different time points post‐treatment, with or without probes. The mice were finally euthanized and their major organs were placed into 4% paraformaldehyde (PFA) for histological examination.

### Hepatic and Renal Function Studies

Blood (≈500 µL) was collected via the fundus venous plexus 24 h after SAH induction, and stored in an ice box to prevent clotting prior to centrifugation at 3500 rpm for 20 min. Serum samples (200 µL) were used to measure total protein (TP), albumin (ALB), alanine aminotransferase (ALT), blood urea nitrogen (BUN), carbon dioxide (CO_2_), creatinine (CREA), cystatin C (CYSC), and urea (UREA) using a Roche Cobas E602 electrochemiluminescence immunoassay analyzer.

### Neurological Scoring and Brain Imaging

Neurological score were determined 24 h post‐surgery prior to euthanization and brain tissue harvesting (Table S1, Supporting Information).^[^ ^49^ ^]^ High resolution photographic images of the brains were used for SAH grading, according to a conventional grading system.^[^ ^50^ ^]^ The basal brain, including the brain stem, was divided into six segments. Each segment was assigned a grade from 0 to 3 depending on the amount of SAH as follows: 0, no SAH; 1, minimal SAH; 2, moderate SAH with recognizable arteries; and 3, SAH covering the cerebral arteries. The animals were assigned a total score ranging from 0 to 18 by summing the scores from all six segments.

### NIR Fluorescence Imaging Following SAH Induction

NIR fluorescence images were acquired 24 h after SAH using a Bruker MI SE 721 instrument. Two imaging steps carried out in sequence: 470 nm × *600 nm with an exposure time of 60 s (excitation wavelength: 470 nm; emission wavelength: 600 nm; acquisition time: 60 s) and white light with an exposure of time of 0.175 s. Fluorescence imaging and quantification were performed using the above‐mentioned instrument under the same conditions.

### MRI Following SAH Induction

MRI was performed 24 h after SAH using a 7.0‐T Varian MR scanner (Varian Inc, Palo Alto, USA) with T2* gradient‐echo acquisition sequences, with a 20 × 20 mm field of view, 256 × 256 mm matrix, and 25 coronal slices (0.5 mm thick). The animals were categorized into five grades as previously described.^[^ ^14^ ^]^

### Histology

20 mm coronal cryostat sections of the main organs were used for H&E stained. Histology was performed by a pathologist blinded to the experimental groups.

### Statistical Analysis

All statistical data are expressed as the mean ± standard deviation (SD) as indicated. Statistical comparisons between two groups were determined using unpaired two‐tailed Student's t‐tests. Difference among multiple groups were determined by One‐way ANOVA. A *p*‐value of 0.0001 was selected as the level of significance, and the data were classified according to their *p*‐values and denoted by **** *p* < 0.0001; “ns” indicated of none significance with *p* > 0.05. Statistical analyses were performed in GraphPad Prism 8 software using statistical tests indicated in the figure legends.

## Conflict of Interest

The authors declare no conflict of interest.

## Supporting information

Supplemental Movie 1

## Data Availability

The data that support the findings of this study are available from the corresponding author upon reasonable request.
